# Dietary Supplementation with an Extract of *Aloysia citrodora* (Lemon verbena) Improves Sleep Quality in Healthy Subjects: A Randomized Double-Blind Controlled Study

**DOI:** 10.3390/nu16101523

**Published:** 2024-05-18

**Authors:** Silvia Pérez-Piñero, Juan Carlos Muñoz-Carrillo, Jon Echepare-Taberna, Macarena Muñoz-Cámara, Cristina Herrera-Fernández, Ana I. García-Guillén, Vicente Ávila-Gandía, Pau Navarro, Nuria Caturla, Jonathan Jones, Francisco Javier López-Román

**Affiliations:** 1 Faculty of Medicine, UCAM Universidad Católica San Antonio de Murcia, Carretera de Guadalupe s/n, 30107 Murcia, Spain; sperez2@ucam.edu (S.P.-P.); echepare@ucam.edu (J.E.-T.); mmunoz5@ucam.edu (M.M.-C.); cherrera@ucam.edu (C.H.-F.); igarcia53@ucam.edu (A.I.G.-G.); vavila@ucam.edu (V.Á.-G.); jlroman@ucam.edu (F.J.L.-R.); 2 Monteloeder s.l., Miguel Servet 16, 03203 Elche, Spain; paunavarro@monteloeder.com (P.N.); nuriacaturla@monteloeder.com (N.C.); jonathanjones@monteloeder.com (J.J.); 3 Primary Care Research Group, Biomedical Research Institute of Murcia (IMIB-Arrixaca), 30120 Murcia, Spain

**Keywords:** sleep disorders, insomnia, *Aloysia citrodora*, lemon verbena, stress, melatonin, cortisol

## Abstract

Seventy-one healthy subjects with sleep disturbances participated in a randomized, double-blind controlled trial in which dietary supplementation with an extract of *Aloysia citrodora* (lemon verbena) (*n* = 33) or placebo (*n* = 38) was administered for 90 days. There were between-group differences in favor of the experimental group in the visual analogue scale (VAS) for sleep quality (6.5 ± 1.6 vs. 5.5 ± 2.1, *p* = 0.021) as well as in the overall score (5.8 ± 2.4, *p* = 0.008) and scores for sleep latency (1.6 ± 1.0 vs. 1.9 ± 0.7, *p* = 0.027) and sleep efficiency (84.5 ± 12.8 vs. 79.8 ± 13.6, *p* = 0.023) in the Pittsburgh Sleep Quality Index (PSQI). Sleep-related variables (latency, efficiency, wakefulness after sleep onset, awakenings) assessed by actigraphy also showed better scores in the experimental group (*p* = 0.001). Plasma nocturnal melatonin levels also increased significantly in the experimental group (199.7 ± 135.3 vs. 174.7 ± 115.4 pg/mL, *p* = 0.048). Changes in anthropometric parameters and physical activity levels were not found. In summary, a dietary supplement of lemon verbena administered for 3 months was associated with a significant improvement in sleep quality as compared with placebo in a population of healthy subjects with sleep problems.

## 1. Introduction

Sleep problems are one of the most common complaints in medical practice. Non-restorative or inadequate sleep can interfere with normal functioning in the physical, mental, and social spheres of life [1]. Sleep disorders may have a profound impact on overall health and quality of life, which in turn are associated with high odds of multimorbidity, including psychiatric and non-psychiatric diseases [2,3,4,5,6]. Although disturbed sleep is easy to diagnose with many self-assessment instruments, sleep disorders are still under-recognized and undertreated in a substantial number of patients, particularly those presenting to physicians with other comorbid medical diseases [7,8] or in many patients who experience sleep problems and do not report them to their doctors [9]. There is a complex interplay with interactive and bidirectional relationships between insomnia and many psychiatric disorders, with the severity of sleep disturbance showing a correlation with the severity of mental illness [4]. However, in clinical practice, clinicians are faced with the challenge of whether to consider sleep disturbances a possible epiphenomenon that will disappear once the primary mental disorder is treated or a valid stand-alone clinical entity [9].

Sleep problems and stress are two conditions which are strongly associated and appear to be pathophysiologically integrated, as the occurrence of stress increases the risk of insomnia, insomnia exacerbates stress, and the coexistence of both conditions has a negative influence on their prognosis [10,11]. Mental stress and poor sleep quality are interlinked outcomes among university and college students in academic settings [12]. Also, it has been shown that healthy individuals and good sleepers are prone to experience situational sleep problems and insomnia under stressful life conditions [13].

Symptomatic treatment of insomnia includes sleep hygiene combined with pharmacological modalities, especially benzodiazepines [14,15]. However, sleep medications improve short-term outcomes but have significant adverse effects and may be addictive. Non-pharmacological therapies have been extensively advocated in the management of different psychiatric and psychological symptoms of stress, particularly to overcome the negative side effects of conventional pharmacotherapy. 

Complementary and alternative medicine strategies based on dietary supplements and popular herbal remedies that are able to improve symptoms of either stress or sleep problems have progressively gained attention, particularly as multimodal interventions used by subjects seeking adjunct non-pharmacological natural therapies [16,17,18]. A combination of hemp oil, calamari oil, and broccoli has been shown to decrease the intensity of pain and improve sleep quality in adults with chronic pain seeking chiropractic care [19]. Among the potential herbs available, *Aloysia citrodora* Paláu (*Lippia citrodora* Kunth), commonly known as ‘lemon verbena’, has shown multiple biological activities, including antioxidant, anxiolytic, neuroprotective, anticancer, aesthetic, antimicrobial, and sedative effects, which have mainly been attributed to verbascoside, an abundant polyphenol found in lemon verbena leaves [20,21,22]. The mechanisms through which lemon verbena exerts its beneficial properties involve binding of the GABA-A receptor, modulation of cAMP and calcium channels, and increased expression of brain-derived neurotrophic factor (BDNF), serotonin, noradrenaline, and dopamine [23,24,25,26]. 

In relation to the potential anxiolytic and sedative effects of the plant, a recent study in 40 subjects with anxiety and sleep problems randomly assigned to supplementation with an extract of lemon verbena (*Lippia citrodora*) or placebo for 8 weeks followed by a 4-week washout period showed that subjects assigned to the intervention group experienced lower stress levels and reported sleeping better [27]. The pharmacokinetic profile of lemon verbena extract containing 25% verbascoside was characterized in an experimental model of male Wistar rats, with a C_max_ at 20 min after administration of the extract, and no signs of toxicity observed at a verbascoside dosage of 500 mg/kg [28].

The present clinical trial was conducted to add further evidence of the efficacy of *Aloysia citrodora* extract in alleviating poor sleep quality in a population of healthy subjects who received dietary supplementation with a nutraceutical formulation of lemon verbena or placebo for 90 days. Also, the effect of the extract on melatonin production was elucidated in the current study.

## 2. Materials and Methods

### 2.1. Study Design and Participants

A randomized, double-blind, placebo-controlled and single-center clinical trial with two parallel arms was carried out at the Health Sciences Department of Universidad Católica San Antonio de Murcia (UCAM) in Murcia, Spain. The study period was from 4 April to 27 July 2023. The primary objective of this study was to assess the effect of a nutraceutical formulation of lemon verbena consumed for 90 days on improving sleep quality in healthy individuals who reported sleep problems as their major complaint. Secondary objectives included evaluating changes in plasma cortisol levels and nocturnal melatonin levels associated with the use of the experimental product and assessing its safety.

Participants were recruited through study advertisements publicized via mass media, social networks, and e-mail lists of the UCAM research institution. Eligibility included subjects of both sexes, aged 18 years or older, with poor sleep quality assessed using the Pittsburgh Sleep Quality Index (PSQI), moderate level of anxiety assessed by the State-Trait Anxiety Inventory (STAI), and able to complete the study procedures. Participants were excluded if at least one of the following criteria were fulfilled: severe or terminal illness; body mass index (BMI) > 32 kg/m^2^; cognitive impairment due to organic diseases (e.g., Parkinson’s disease, Alzheimer’s disease, Huntington’s disease); use of drugs that may affect cognitive performance or sleep quality, such as anticonvulsants, barbiturates, benzodiazepines, antidepressants, neuroleptics, alcohol and drugs of abuse; known allergy to any of the study components; pregnant and breastfeeding women; participation in another clinical trial that included blood sampling or a dietary intervention; and inability to provide informed consent.

The study protocol was approved by the Ethics Committee of Universidad Católica San Antonio de Murcia (code CE032302; approval date 30 March 2023) (Murcia, Spain) and was registered in ClinicalTrials.gov (NCT06154629). All participants provided written informed consent.

### 2.2. Investigational Product

The study product was a purified extract of lemon verbena (*A. citrodora*), standardized at a minimum of 24% verbascoside and 28% of total phenylpropanoids, provided by Monteloeder SL (Alicante, Spain). The extraction process was the same as that used in a previous study [27]. Briefly, dried leaves were mixed with at least 70% ethanol and kept macerated at less than 80 °C for 2 h. A silica–cellulose filter of 1 µm was used to eliminate insoluble particles and the filtered mixture was dried in a vacuum at 60–80 °C. Then, the extract was dissolved at a concentration of 1 mg/mL for high-performance liquid chromatography (HPLC) analysis (1260 Infinity II LC System, Agilent Technologies Spain, S.L., Las Rozas, Madrid, Spain) at 330 nm. Other technical conditions were the use of acetic acid and acetonitrile as mobile phases, an injection volume of 20 µL, and a flow rate of 1 mL/min. The HPLC compounds detected included luteolin-7-diglucuronide (retention time [RT] 7.013 min), verbascoside (RT 15.040 min), diosmetin-7-O-diglucuronide (RT 15.720 min), chrysoeriol-7-diglucuronide (RT 15.987 min), isoverbascoside (RT 16.413 min), forsythoside A (RT 17.253 min), eukovoside (RT 20.027 min), acacetin-7-diglucuronide (RT 25.667 min), and martynoside (RT 27.427 min). 

Each capsule contained 400 mg of lemon verbena and 150 mg of an excipient (cellulose microcrystalline). Placebo capsules contained cellulose microcrystalline and maltodextrin and had the same organoleptic properties as the investigational product. All participants were instructed to take 1 capsule per day of the assigned supplement, 1 h before sleeping, for 90 consecutive days. 

### 2.3. Randomization and Study Procedures

Participants were randomized to an intervention (experimental) group or a placebo group by an independent center using a simple randomization procedure (1:1) in Epidat software version 4.1.

The dietary supplement was provided at the baseline visit and at the mid-study visit (day 45) and subjects were required to consume at least 80% of the capsules, so that only 18 capsules could be left in total corresponding to 18 days out of 90 days of consumption. Subjects were advised not to introduce changes in their dietary habits or levels of physical activity. The use of any new medication was to be reported to the principal investigator. 

This study included a screening visit, a baseline visit (visit 1), a mid-study visit at 45 days (visit 2), and a final visit at 90 days (visit 3) at the end of this study. The screening visit took place within ± 10 days prior to the baseline visit, in which the inclusion criteria were checked and written informed consent was obtained. Patients were also randomized to one of the study groups.

At the baseline visit (visit 1), the data recorded were as follows: medical history; physical examination; vital signs; body weight and height; quality of sleep according to the PSQI questionnaire, the VAS score, and actigraphy-based sleep parameters; level of stress using the Perceived Stress Scale (PSS) and anxiety using the STAI questionnaire; body composition by bioelectrical impedance analysis (BIA); and level of physical activity. Laboratory tests included plasma cortisol levels, nocturnal melatonin levels, and standard hematological and biochemical parameters. The dietary supplement was provided at the baseline visit.

At the mid-study visit (45 days, visit 2), study procedures of the baseline visit were repeated, except for laboratory tests. The remaining dietary product was collected and compliance was calculated. Subjects received the assigned product for additional 45 days of treatment and adverse events (AEs) were registered. At the final visit (90 days, visit 3), the same study procedures as those of the baseline visit were performed. The investigational product was collected and compliance was calculated. AEs were recorded. 

### 2.4. Study Variables

A 10 cm unnumbered VAS scale was used to assess sleep quality in the past month, where 0 was ‘very poor sleep quality’ and 10 ‘very good sleep quality’. Scores were determined by measuring the distance between the ‘very poor sleep quality’ anchor and the subject’s mark on the 10 cm line. VAS scores < 5 indicated poor–moderate sleep quality, whereas scores > 6 indicated moderate–good sleep quality.

The PSQI is a short self-report questionnaire and the most widely used subjective measure of sleep quality over an interval of 1 month. The instrument measures seven dimensions from 0 (best) to 3 (worst). These seven components can be broadly categorized into sleep efficiency factors (sleep quality, sleep latency, sleep duration, and habitual sleep efficiency), to which sleep-disturbing factors (sleep disturbance, use of sleep medications, and daytime disturbance) can be added. Adding up the average scores of the seven components gives a global PSQI score from 0 to 21. Higher scores indicate worse sleep quality. In this study, an overall PSQI score > 5 was required. A Spanish-validated version of the PSQI was administered [29].

Sleep quality was measured for 24 h using a wrist-worn accelerometer (ActiGraph wGT3X-BT accelerometer, ActiGraph, Pensacola, FL, USA) during 3 days of the week and 1 day of the weekend, at the baseline visit and after 45 and 90 days of consumption of the dietary product. The following variables were recorded: sleep latency, sleep efficiency, total time in bed, total sleep time, wakefulness after sleep onset, number of awakenings, and average number in minutes of awakenings.

The STAI questionnaire measures state (STAI-state) and trait (STAI-trait) anxiety based on 20 questions for each domain. Scores can vary between 0 and 60, with higher scores indicating greater anxiety levels. Scores 0–9 indicate normal or no anxiety, 10–18, mild-to-moderate anxiety, 19–29, moderate-to-severe anxiety; and 30–60, severe anxiety. A Spanish-validated version was used [30].

The PSS scale includes 14 items measuring the frequency or extent of a certain stress-signaling event occurrence of a 5-point scale. Total perceived stress level scores range between 0 and 56, with higher scores indicating greater perceived stress over the previous month. A Spanish-validated version was used [31].

A whole-body bioimpedance analyzer (Tanita BC-420MA, Tanita Corp., Tokyo, Japan) was used to determine corporal composition (weight, body mass index [BMI], fat mass [expressed in kg], percentage of fat mass, and muscle mass [expressed in kg]). The level of physical activity was evaluated with the accelerometer used to assess sleep quality, with results expressed as metabolic equivalents (METs).

Plasma cortisol levels were measured in blood samples taken early in the morning and nocturnal melatonin levels in samples taken late in the evening. Blood samples for plasma cortisol measurement were collected between 6:00 and 7:00 a.m. in fasting conditions, and cortisol levels were determined using a commercial ELISA kit (QuicKey Pro Human Cortisol ELISA Kit, Elabscience^®^, Madrid, Spain) following the manufacturer’s instructions. Blood samples for measurement of plasma melatonin levels were collected at night between 10:00 and 11:00 p.m. after the subjects had been in a dimly lit room for 20–30 min. Analyses were performed using a commercial ELISA kit (Human MET (Melatonin) ELISA Kit, bioNova Científica, S.L., Madrid, Spain) following the manufacturer’s instructions. Results are expressed as pg/mL. Safety data included blood pressure recording and laboratory analyses. Standard hematological (hemogram) and biochemical parameters (renal and liver function tests) were measured.

### 2.5. Study Endpoints

The primary endpoint of this study was the change from baseline to the end of study (90 days) in the quality of sleep measured by VAS in the experimental arm (lemon verbena) as compared with the placebo arm. Secondary endpoints were changes in sleep quality measured by the PSQI and actigraphy as well as changes in PSS, STAI, and plasma cortisol and nocturnal melatonin levels after 90 days of consumption of the dietary supplement. Safety endpoints were anthropometric variables, level of physical activity, vital signs, laboratory values, and AEs.

### 2.6. Statistical Analysis

Data of all participants who met the eligible criteria and completed the 90-day study period were analyzed. Categorical data are expressed as frequencies and percentages, and continuous data as means and standard deviations (±SDs). Differences in the distribution of variables between the experimental and control groups were analyzed with the chi-square (χ^2^) test for qualitative variables and Student’s *t* test for quantitative variables. Analysis of variance (ANOVA) for repeated measures was used to assess the change of variables corresponding to each group throughout the study period. The subject factor included data at baseline, at the mid-study visit (45 days), and at the final visit (90 days) and the between-subject factor for paired data included the product administered, that is lemon verbena or placebo. Tukey’s or Bonferroni’s correction was applied for post hoc analyses. A *p* < 0.05 was considered statistically significant. Data were analyzed with the SPSS version 25.0 (IBM Corp., Armonk, NY, USA) software program.

## 3. Results

### 3.1. Participants

**Figure 1:**
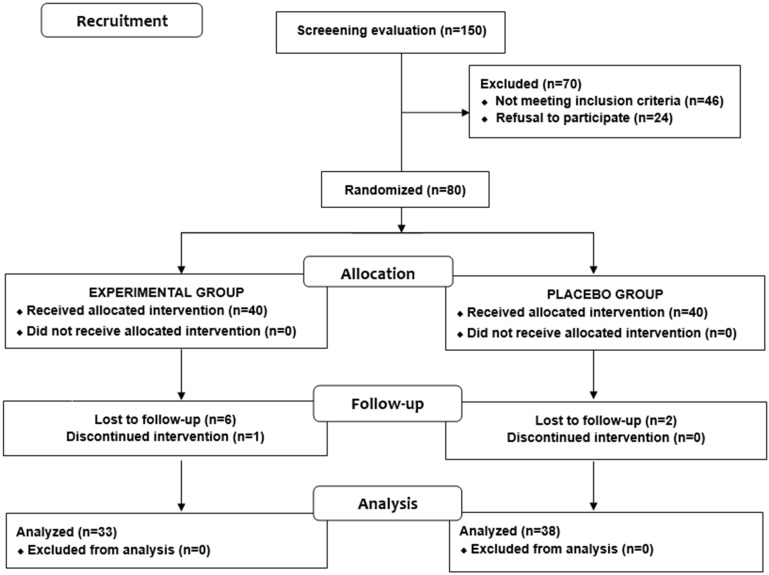
Distribution of the participants in the two study groups.

Of a total of 150 subjects who were initially selected for this study, 80 were eligible. A total of 70 subjects were excluded; 46 because the inclusion criteria were not met and the remaining 24 because they refused to participate. Of the 80 eligible subjects, 40 were randomized to the experimental (lemon verbena) group and 40 to the placebo group. During the intervention period, nine subjects were lost to follow-up, seven from the experimental group and two from the control group. The final study population included 71 subjects (33 in the experimental group and 38 in the placebo group). A flow chart distribution of the participants is shown in Figure 1.

Regarding the baseline characteristics of participants, the mean age was 29.5 ± 11.2 years and the mean weight was 70.8 ± 15.6 kg. The mean VAS score of sleep quality was 3.7 ± 1.7, and the mean systolic and diastolic blood pressure 114.5 ± 12.0 and 74.6 ± 7.4 mmHg, respectively. Differences in baseline data between the study groups were not found (Table 1).

### 3.2. Sleep Quality

#### 3.2.1. VAS Scores

The changes in VAS sleep quality scores over the previous month as rated by subjects assigned to the experimental and placebo groups throughout the study are shown in Table 2. Sleep quality improved significantly in both study groups in the comparison between values recorded at baseline and at the mid-term and final visits. However, the use of the interventional product over 90 days was associated with a statistically significant greater improvement in the quality of sleep as compared with placebo (*p* = 0.021). 

#### 3.2.2. PSQI Scores

After 45 days of consumption of the assigned dietary supplement, there was an improvement in the overall score and all components of the PSQI questionnaire as compared with the baseline. At the end of the study period (90 days), the comparison of the overall score, sleep latency, and sleep efficiency between the experimental and the placebo groups showed significant differences (*p* < 0.05) in favor of the experimental group. Sleep latency also improved significantly between visit 2 at 45 days and visit 3 at 90 days in the experimental group (*p* = 0.042). Subjects assigned to the experimental group showed statistically significant differences as compared with placebo over the study period in the overall score of the PSQI questionnaire as well as in the domains of sleep latency and sleep efficiency (Table 3).

#### 3.2.3. Actigraphy

Results of actigraphy sleep studies are shown in Table 4. Participants assigned to the experimental group as compared to those administered with placebo had statistically significant improvements in the four domains of sleep latency, sleep efficiency, wakefulness after sleep onset, and mean number of awakenings at the final visit on day 90. Moreover, statistically significant between-group differences for these four sleep domains were observed at the final visit on day 90. In the remaining domains of total time in bed, total sleep time, and number of awakenings, differences between the study groups as well as within each group during the study period were not detected.

### 3.3. Perceived Stress and Anxiety

As shown in Table 5, PSS total scores showed a decreasing trend over the study period, which was of greater magnitude in the experimental group (mean decrease of 9.1 points) as compared with the placebo group (mean decrease 5.8 points), although between-group differences were not statistically significant. In relation to anxiety levels, subjects assigned to the experimental group showed a statistically significant decrease in scores in the STAI-state scale as compared with placebo (*p* = 0.037), whereas changes in the STAI-trait scale were not observed (Table 5). 

### 3.4. Plasma Cortisol and Nocturnal Melatonin Levels

As shown in Table 6, plasma cortisol levels did not change in any of the study groups, whereas plasma nocturnal melatonin levels increased significantly in the experimental arm as compared with placebo.

### 3.5. Anthropometric Variables and Level of Physical Activity

Changes in anthropometric variables measured by BIA and levels of physical activity were not statistically significant in any of the study groups over the 90 days of administration of the dietary supplement (Table 7).

### 3.6. Compliance and Safety 

All participants consumed at least 80% of the study product. The maximum number of capsules returned was 12, with compliance ranging between 100% and 86.6%. Changes in systolic and diastolic blood pressure and heart rate during the study period were not observed. Also, the results of laboratory tests remained within the normal ranges (Table S1). AEs related to the consumption of the study product were not observed. 

## 4. Discussion

In the present study, in a group of 71 of adult subjects with low sleep quality (mean VAS score of 3.6 and mean PQSI score of 10.4 at baseline), the administration of a dietary supplement of lemon verbena extract for 90 days was associated with significant improvement in the quality of sleep as compared to placebo. 

Improvements in the quality of sleep in the experimental group were noted in all sleep-related variables measured with the three study methods: the VAS score, the PSQI questionnaire, and the actigraphy device. In relation to stress-related complaints, subjects assigned to the experimental group also showed improvements in the overall score of the PSS scale, which were of a greater magnitude as compared with placebo (non-significant differences), as well as a significant amelioration of the STAI-state score. Interestingly, melatonin levels significantly increased in subjects consuming the dietary supplement.

Since ancient times, plants have played a major role as sources of medicine for humans, and herbal plant extracts continue to be a widely accepted complementary medical option. The use of herbal medicinal products and supplements has increased tremendously over the past three decades, with no less than 80% of people worldwide relying on them, especially in primary healthcare [32]. Botanical-based formulas have been integrated as effective and safe approaches for a large variety of complaints, especially those that are common in the general population, such as sleep problems and feelings of stress. In light of this, the World Health Organization (WHO) has developed research guidelines for the rational use and further development of herbal medicines, which should be supported by appropriate scientific studies of these products [33]. 

*A. citrodora* (lemon verbena), a species of flowering plant of the verbena family Verbenacea, is native to South America, but it is cultivated in many other parts of the world, including the Middle East and the Mediterranean region [34]. Among the pleomorphic properties of the plant, mostly attributed to the polyphenol verbascoside [20], a few clinical studies have examined the sedative effects of lemon verbena for improving sleep disorders and reducing anxiety symptoms. In a randomized, double-blind and placebo-controlled study, Afrasiabian et al. [35] reported the results of the administration of 10 cc of a syrup of *A. citrodora* (total essential oil content of the product 1.66 mg/10 mL and flavonoid quercetin 3.22 mg/10 mL) or placebo one hour before bedtime for 4 weeks in subjects with insomnia. All participants had a score > 5 of the PSQI questionnaire. The final analysis included 47 subjects in the intervention group and 43 in the placebo group. The main findings of this study were significant improvements in four components of the PSQI questionnaire (sleep latency, habitual sleep efficiency, daytime dysfunction, and subjective sleep quality) and also in the overall score of the Insomnia Severity Index (ISI) questionnaire, which indicated better sleep quality in the *A. citrodora* group. A benzodiazepine-like effect on the GABA receptor has been suggested as a plausible mechanism of action of *A. citrodora* in experimental studies [25,36]. 

In our study, after 90 days of supplementation, we found significant differences in the overall score of the PSQI and in the subscales of sleep latency and sleep efficiency, with improvements already observed in data analysis at the mid-study visit, with better sleep quality in subjects administered with lemon verbena. The beneficial effects of supplementation with lemon verbena on sleep parameters were also found in sleep analysis by actigraphy. In this case, not only did sleep latency and sleep efficiency improve significantly in the experimental group, but the results obtained for wakefulness after sleep onset and duration of awakenings were also significantly more favorable as compared with the placebo group. These additional benefits could be due to the specific characteristics of the lemon verbena extract used, which was standardized to contain at least 24% verbascoside.

The tranquilizing effect of lemon verbena have also been reported by others. In a randomized single-blind aromatherapy study in women undergoing a cesarean section, the administration of three inhaled drops of lemon verbena essential oil before surgery as compared to distilled water reduced preoperative anxiety [37]. Martínez-Rodríguez et al. [27] conducted a randomized double-blind controlled study to assess the effect of dietary supplementation with *Lippia citrodora* vs. placebo in subjects with sleep problems and anxiety symptoms who received this supplement over an 8-week study period. The results of this study are similar to our findings, with a reduction in perceived stress and improved sleep quality and with a stronger effect on sleep quality in women. A previous study by these authors in two groups of 14 volunteers, each consuming an extract of lemon verbena or placebo during 21 days, the results of the Profile of Mood States (POMS) showed reduced values in the subscales of tension–anxiety, anger–hostility, and fatigue–inertia at the end of the study compared to placebo [38].

The consumption of lemon verbena extract over 90 days was associated not only with better sleep quality, but also with a significant increase in nocturnal melatonin levels. These findings are consistent with the restoration of nocturnal melatonin secretion in patients with sleep disorders treated with sleep medications [39,40]. As far as we are aware, this is the first study to demonstrate, at least clinically, that lemon verbena can increase the production/secretion of melatonin at night. Other studies have determined the presence of melatonin in herbal products and supplements, but quantification of changes in plasma melatonin concentrations before and after dietary supplementation with natural herbal extracts has not been reported. Padumanonda et al. [41] determined the melatonin content in extracts of seven edible herbs used as sleeping aids in Thai traditional medicine and reported 43.2 ng/g of dry sample weight for *Boehmeria ramiflora*, 26.3 ng/g for *Sesbandia glandiflora*, 21.4 ng/g for *Mormodica charantia*, 10.5 ng/g for *Senna tora*, and 8.7 ng/g for *Sesbania sesban*, with *Piper nigrum* having the highest melatonin content (1092.7 ng/g of dry sample weight). Herrera et al. [42] determined the melatonin content in herbal infusions of 19 commercial medicinal plants, with the highest levels of melatonin of 4.6–6.0 ng/mL (corresponding to 1.3–1.5 µg/cup) in chamomile, green tea, valerian, and hawthorn. A systematic review characterized melatonin signaling networks in plants [43] and an analysis of 31 commercial supplements using ultraperformance liquid chromatography with electrochemical detection showed a large variability, with melatonin content ranging from −83% to +478% of the labeled content [44]. 

Analysis of plasma cortisol levels was unrevealing in our study, without differences between baseline and 90-day values in any of the study groups. In the study of Martínez-Rodríguez et al. [27], cortisol levels showed a 15.6% decrease after 2 months of supplementation with lemon verbena extract, which coincided with decreased perceived stress reported in this study. Lower baseline values of plasma cortisol levels in the present study may account for the differences as compared with the study of Martínez-Rodríguez et al. [27].

All changes in study variables were unrelated to the effect of anthropometric variables or physical activity levels since changes in these parameters between baseline and the end of the study did not occur. Moreover, all participants showed a good adherence to the study products. The investigational supplement of lemon verbena was well tolerated and safe. 

The present results should be interpreted while taking into account some of this study’s limitations, particularly the limited sample size and the 90-day duration of dietary supplementation. Although it was emphasized that participants should maintain their dietary habits over the course of this study, strict control of dietary intake was not performed. Plasma cortisol is known to follow a diurnal cycle with highs/lows and several intermittent pulses throughout the day, but the results of plasma cortisol levels obtained in the present study should be considered while taking into account the limitation associated with single-point quantification.

## 5. Conclusions

The administration of a dietary supplement of *Aloysia citrodora* (lemon verbena) extract for 90 days in healthy volunteers with sleep problems resulted in a significant improvement in sleep quality as compared with placebo. Improvements were also apparent at the mid-study visit after 45 days of supplementation. Melatonin levels also significantly increased in the experimental group. However, further randomized controlled studies with a larger study population and a more prolonged duration of supplementation are necessary to confirm these findings.

## Figures and Tables

**Table 1:** Baseline characteristics of the study population.

Variables	Placebo (*n* = 38)	Experimental (*n* = 33)	Total (*n* = 71)
Age, years	29.6 ± 10.9	29.4 ± 11.6	29.5 ± 11.2
Weight, kg	70.8 ± 15.6	68.2 ± 12.7	69.6 ± 14.3
VAS score, sleep quality	3.8 ± 2.0	3.5 ± 1.4	3.7 ± 1.7
Systolic BP, mmHg	112.4 ± 10.3	117.0 ± 13.4	114.5 ± 12.0
Diastolic BP, mmHg	74.8 ± 7.6	74.2 ± 7.3	74.6 ± 7.4

VAS: visual analogue scale, BP: blood pressure. Data as mean ± standard deviation.

**Table 2:** Changes in visual analogue scale (VAS) scores of sleep quality in the two study groups.

Study Group	Visit 1 Baseline	Visit 2 Mid-Study (45 days)	Within-Group Differences Visits 1 vs. 2 *p* Value	Visit 3 Final (90 days)	Within-Group Differences Visits 1 vs. 3 *p* Value	Within-Group Differences Visits 2 vs. 3 *p* Value	Between-Group Differences *p* Value
Placebo (*n* = 38)	3.8 ± 2.0	5.0 ± 2.0	0.004	5.5 ± 2.1	0.001	0.057	0.021
Experimental (*n* = 33)	3.5 ± 1.4	5.8 ± 1.8 *	0.001	6.5 ± 1.6 *	0.001	0.010	

Data as mean ± standard deviation; * *p* < 0.05 for between-group comparisons at the 45-day and 90-day study visits.

**Table 3:** Changes of scores in the different components of the PSQI questionnaire in the two study groups.

Variables	Visit 1 Baseline	Visit 2 Mid-Study (45 days)	Within-Group Differences Visits 1 vs. 2 *p* Value	Visit 3 Final (90 days)	Within-Group Differences Visits 1 vs. 3 *p* Value	Within-Group Differences Visits 2 vs. 3 *p* Value	Between-Group Differences *p* Value
Overall score							
Placebo (*n* = 38)	10.2 ± 3.0	7.7 ± 2.9	0.001	7.4 ± 2.9	0.001	1.000	0.008
Experimental (*n* = 33)	10.6 ± 2.1	6.7 ± 2.7	0.001	5.8 ± 2.4 *	0.001	0.074	
Sleep quality							
Placebo (*n* = 38)	2.0 ± 0.5	1.2 ± 0.6	0.001	1.2 ± 0.6	0.001	1.0	0.486
Experimental (*n* = 33)	2.1 ± 0.4	1.3 ± 0.7	0.001	1.1 ± 0.6	0.001	0.311	
Sleep latency							
Placebo (*n* = 38)	2.3 ± 0.8	1.9 ± 0.7	0.016	1.9 ± 0.7	0.029	1.0	0.027
Experimental (*n* = 33)	2.4 ± 0.7	1.9 ± 0.9	0.001	1.6 ± 1.0 *	0.001	0.042	
Sleep duration							
Placebo (*n* = 38)	2.1 ± 0.8	1.7 ± 0.9	0.019	1.5 ± 0.9	0.001	0.546	0.722
Experimental (*n* = 33)	1.9 ± 0.8	1.4 ± 1.0	0.003	1.2 ± 1.0	0.001	0.457	
Sleep efficiency, %							
Placebo (*n* = 38)	76.2 ± 17.9	78.8 ± 12.0	0.800	79.8 ± 13.6	0.366	1.0	0.023
Experimental (*n* = 33)	72.3 ± 12.4	81.5 ± 14.0 *	0.001	84.5 ± 12.8 *	0.001	0.439	
Disturbances, number							
Placebo (*n* = 38)	12.8 ± 3.7	10.1 ± 3.6	0.001	9.4 ± 3.3	0.001	0.488	0.382
Experimental (*n* = 33)	12.8 ± 4.0	9.0 ± 3.8	0.001	8.5 ± 3.8	0.001	1.0	

Data as mean ± standard deviation; * *p* < 0.05 for between-group comparison at 45-day and 90-day study visits.

**Table 4:** Results of sleep evaluation by actigraphy in the two study groups.

Variables	Visit 1 Baseline	Visit 2 Mid-Study (45 days)	Within-Group Differences Visits 1 vs. 2 *p* Value	Visit 3 Final (90 days)	Within-Group Differences Visits 1 vs. 3 *p* Value	Within-Group Differences Visits 2 vs. 3 *p* Value	Between-Group Differences *p* Value
Sleep latency, min							
Placebo (*n* = 38)	3.0 ± 0.9	3.2 ± 0.8	0.459	3.2 ± 0.8	0.925	1.0	0.001
Experimental (*n* = 33)	3.7 ± 0.9	3.3 ± 0.9	0.091	2.9 ± 0.8 *	0.001	0.006	
Total time in bed, min							
Placebo (*n* = 38)	436.9 ± 49.0	424.0 ± 63.5	0.556	422.1 ± 51.2	0.284	1.0	0.985
Experimental (*n* = 33)	432.2 ± 40.0	418.6 ± 51.5	0.586	418.8 ± 61.7	0.474	1.0	
Total sleep time, min							
Placebo (*n* = 38)	400.4 ± 48.7	384.5 ± 58.5	0.200	386.4 ± 49.9	0.243	1.0	0.428
Experimental (*n* = 33)	390.0 ± 36.4	383.4 ± 47.5	1.0	390.9 ± 57.6	1.0	1.0	
Sleep efficiency, %							
Placebo (*n* = 38)	91.6 ± 2.9	90.7 ± 3.5	0.335	91.5 ± 3.4	1.0	0.458	0.001
Experimental (*n* = 33)	90.3 ± 2.9	91.6 ± 2.9 *	0.050	93.3 ± 2.5 *	0.001	0.015	
Wakefulness after sleep onset, min							
Placebo (*n* = 38)	34.1 ± 13.0	36.6 ± 15.3	0.385	35.0 ± 13.5	1.0	0.843	0.001
Experimental (*n* = 33)	36.3 ± 10.5	29.1 ± 8.3 *	0.001	28.7 ± 9.2 *	0.001	1.0	
Number of awakenings							
Placebo (*n* = 38)	15.8 ± 5.1	16.1 ± 5.7	1.0	15.9 ± 4.3	1.0	1.0	0.606
Experimental (*n* = 33)	15.8 ± 4.0	15.3 ± 5.2	1.0	14.8 ± 4.8	0.642	1.0	
Awakenings, mean number of min							
Placebo (*n* = 38)	2.2 ± 0.6	2.3 ± 0.8	0.128	2.2 ± 0.6	1.0	0.322	0.001
Experimental (*n* = 33)	2.3 ± 0.5	2.0 ± 0.5 *	0.003	2.0 ± 0.6 *	0.006	1.0	

Data as mean ± standard deviation; * *p* < 0.05 for between-group comparison at the 45-day and 90-day study visits.

**Table 5:** Changes in stress levels and anxiety in the two study groups.

Variables	Visit 1 Baseline	Visit 2 Mid-Study (45 days)	Within-Group Differences Visits 1 vs. 2 *p* Value	Visit 3 Final (90 Days)	Within-Group Differences Visits 1 vs. 3 *p* Value	Within-Group Differences Visits 2 vs. 3 *p* Value	Between-Group Differences *p* Value
PSS total score							
Placebo (*n* = 38)	29.4 ± 8.3	27.0 ± 8.9	0.050	23.6 ± 9.0	0.008	0.225	0.347
Experimental (*n* = 33)	31.4 ± 8.5	26.5 ± 7.8	0.001	22.3 ± 7.9	0.001	0.139	
STAI-state, score							
Placebo (*n* = 38)	24.9 ± 11.3	21.9 ± 10.8	0.116	19.8 ± 10.3	0.001	0.063	0.037
Experimental (*n* = 33)	27.4 ± 10.4	22.8 ± 10.2	0.010	17.6 ± 8.7 *	0.001	0.001	
STAI-trait, score							
Placebo (*n* = 38)	27.4 ± 11.6	25.2 ± 11.4	0.050	23.5 ± 12.6	0.001	0.073	0.492
Experimental (*n* = 33)	29.9 ± 11.2	27.4 ± 10.4	0.039	24.6 ± 9.6	0.001	0.002	

PSS: Perceived Stress Scale; STAI: State-Trait Anxiety Inventory. Data as mean ± standard deviation; * *p* < 0.05 for between-group comparison at the 90-day study visit.

**Table 6:** Changes in plasma cortisol and nocturnal melatonin levels in the two study groups.

Variables	Visit 1 Baseline	Visit 3 Final (90 days)	Within-Group Differences Visits 1 vs. 3 *p* Value	Between-Group Differences *p* Value
Cortisol, pg/mL				
Placebo (*n* = 38)	52.1 ± 26.4	51.1 ± 25.0	0.433	0.370
Experimental (*n* = 33)	51.6 ± 13.6	52.9 ± 18.0	0.383	
Nocturnal melatonin, pg/mL				
Placebo (*n* = 38)	182.0 ± 92.2	174.7 ± 115.4	0.485	0.048
Experimental (*n* = 33)	176.1 ± 94.0	199.7 ± 135.3	0.039	

Data as mean ± standard deviation.

**Table 7:** Anthropometric variables and level of physical activity in the two study groups.

Variables	Visit 1 Baseline	Visit 2 Mid-Study (45 Days)	Within-Group Differences Visits 1 vs. 2 *p* Value	Visit 3 Final (90 days)	Within-Group Differences Visits 1 vs. 3 *p* Value	Within-Group Differences Visits 2 vs. 3 *p* Value	Between-Group Differences *p* Value
Weight, kg							
Placebo (*n* = 38)	70.8 ± 15.6	70.6 ± 15.5	0.598	70.5 ± 15.5	0.596	1.0	0.342
Experimental (*n* = 33)	68.2 ± 12.7	68.2 ± 12.6	1.0	67.6 ± 12.4	0.256	0.015	
BMI, kg/m^2^							
Placebo (*n* = 38)	24.9 ± 4.3	24.9 ± 4.4	1.0	24.8 ± 4.3	1.0	1.0	0.438
Experimental (*n* = 33)	24.0 ± 3.1	24.0 ± 3.1	1.0	23.8 ± 3.1	0.229	0.050	
Fat mass, kg							
Placebo (*n* = 38)	21.6 ± 10.3	20.4 ± 9.5	0.001	20.3 ± 9.8	0.002	1.0	0.353
Experimental (*n* = 33)	17.9 ± 6.6	17.2 ± 6.4	0.050	17.2 ± 6.3	0.235	1.0	
Fat mass, %							
Placebo (*n* = 38)	29.1 ± 8.3	27.8 ± 8.1	0.001	27.7 ± 8.5	0.001	1.0	0.488
Experimental (*n* = 33)	25.7 ± 7.6	24.7 ± 7.4	0.001	24.9 ± 7.3	0.050	1.0	
Muscle mass, kg							
Placebo (*n* = 38)	47.0 ± 8.7	47.7 ± 8.7	0.005	47.7 ± 8.8	0.023	1.0	0.154
Experimental (*n* = 33)	48.3 ± 10.1	49.0 ± 10.0	0.003	48.5 ± 9.8	1.0	0.043	
Physical activity, METs							
Placebo (*n* = 38)	1.55 ± 0.27	1.55 ± 0.24	1.0	1.53 ± 0.28	1.0	1.0	0.926
Experimental (*n* = 33)	1.50 ± 0.16	1.51 ± 0.23	1.0	1.49 ± 0.19	1.0	1.0	

BMI: body mass index; METs: metabolic equivalents. Data as mean ± standard deviation.

## Data Availability

Data are available from the corresponding author upon request due to privacy.

## Supplementary Materials

The following supporting information can be downloaded at: https://www.mdpi.com/article/10.3390/nu16101523/s1, Table S1: Evolution of biochemical variables in relation to product safety in the two study groups.
