# Supplementary material for: Dietary Supplementation with an Extract of Aloysia citrodora (Lemon verbena) Improves Sleep Quality in Healthy Subjects: A Randomized Double-Blind Controlled Study
Source: Nutrients. 2024 May 18;16(10):1523. doi: 10.3390/nu16101523 (PMC11123999; doi:10.3390/nu16101523)
Supplement: Supplementary file 1 [file nutrients-16-01523-s001.zip › nutrients-2981129-supplementary.pdf]

Supplementary Table S1. Evolution of biochemical variables in relation to product safety in the two study groups.

		Creatinine (mg/dl)		AST (UI/l)		ALT (UI/l)		GTT (UI/l)	
		**Mean ± SD**	**Abnormal values**	**Mean ± SD**	**Abnormal values**	**Mean ± SD**	**Abnormal values**	**Mean ± SD**	**Abnormal values**
**Placebo**	**V1**	0.9 ± 0.13	S6: 1.28<br>S59: 1.25	18.39 ± 3.96		20.32 ± 9.98		19.86 ± 14.56	S73: 82.3
	**V3**	0.78 ± 0.12		21.24 ± 17.16	S43: 119	17.98 ± 9.3		18.9 ± 16.29	S69: 86.9
**Experimental**	**V1**	0.93 ± 0.15	S33: 1.4	19.32 ± 5.11		19.89 ± 11.35	S30: 60.7	18.02 ± 12.47	S13: 76.5
	**V3**	0.82 ± 0.13		18.77 ± 6		19.34 ± 12.03		16.29 ± 9.87	S13: 63.4

S: Study subject who has had abnormal values
